# Supplementary material for: Thirteen dubious ways to detect conserved structural RNAs
Source: IUBMB Life. Author manuscript; Available in PMC 2024 Jul 10. (PMC11234323; doi:10.1002/iub.2694)
Supplement: supplementary_material [file NIHMS1907110-supplement-supplementary_material.gz › supplemental_material/Table1/CRS.structural.tar.gz!/selected/selected_noRfam/noNUMTs/cmf.M2304242/MyM2304242/R-scape_nhmmer/cmf.M2304242_ext_liftover.hg38.R2R.sto.pdf]

cmf.M2304242_ext_liftover.hg38

5′-○○○○○○○○○○○●R●YUUYUU●GGRCUYUU●GYY●YYUU●○○○○○○○○●●R●●GC●GC●GGYUUCUU●GCYUUCUU●GGR●●CUUYUU●RY●○○○○○○○○○○○○○○○○○○○○○●YYRY●GCYUUCUUGGCUUCUURGC●GC●●Y●GC●GGYUUCURG●YUU●G●●GY●●Y●R●CUUYYU●GYYUU●●●CUU●RYYUY●●○○○○○○○Y●●●CUUCUUGYU●AGYUUGAARGARCC●GARGCRCCRGURCCYUUGGUCUGYACCAGRGUGCCYUUR●U●AC●AGGCUCUURA●●●C●ARCUUGAYGCGG●●GUUGUUCUUCUCCACRUCGUAR
